# Woman

**Published:** 1890-02

**Authors:** 


### WOMAN.

Bishop Fallows, of Chicago, in his sermon before the National W. C. T. U. Convention in New York, said among other things:

Woman represents, and largely is, the conscience and heart of Christendom. Conviction in her has spiritual efficacy. Love kindles judgment, and high purpose is sublimated in passion. More than man she beat down slavery in this country. More than man she is to mold the future of the world. Now, more than ever before, the earth of the prophesy helps the woman, and gives to her immense opportunity. The shining and stimulating spirit in matrons and maidens, to whom was revealed the heavenly Lord, has been a power and a beauty from the beginning—never more than in late years. Their delicate hands hold at this hour, I firmly believe, the lever which must lift the moral and Christian civilization of the world. It is theirs to set in swifter motion the wheels of beryl, vivid with life, which are under the throne. It is theirs to open for the peoples the gates of life. With that intense and exhilerating temper of which we have already felt the blessing, universal among them, and subtly diffused through homes and congregations, the appearing brightness will be as the appearance of the bow in the cloud in the day of rain. For the one thing wanting for the church of our day will at last be supplied—its desire will be equal to its power; its zeal will match its mighty occasions.

